# Operative Hysteroscopy Intravascular Absorption Syndrome Causing Hyponatremia with Associated Cerebral and Pulmonary Edema

**DOI:** 10.5811/cpcem.2019.4.41878

**Published:** 2019-06-04

**Authors:** Marco F. Elegante, Joseph A. Hamera, Jane Xiao, David A. Berger

**Affiliations:** * Oakland University William Beaumont School of Medicine, Department of Emergency Medicine, Royal Oak, Michigan; † Oregon Health Sciences University, Department of Emergency Medicine, Portland, Oregon

## Abstract

Operative hysteroscopy intravascular absorption syndrome is an iatrogenic syndrome caused by absorption of hypo-osmolar distension medium during hysteroscopy, which can lead to rapid hyponatremia with resulting cerebral and pulmonary edema. We present a case of a 47-year-old female who underwent hysteroscopic myomectomy at an outpatient ambulatory surgical center who was brought to the emergency department with dyspnea, hypoxia, and altered mental status. Workup showed hyponatremia with cerebral edema on computed tomography of the head and pulmonary edema on chest radiograph. The patient improved after resuscitation with intravenous saline and supplemental oxygen, and she was discharged home the next day.

## INTRODUCTION

Hysteroscopy has been increasingly common since the 1990s. As this procedure becomes more commonplace in ambulatory surgical centers there will be an increased need for postoperative complications to be evaluated and initially managed in emergency departments (ED). Operative hysteroscopy intravascular absorption (OHIA) syndrome was first described in 1993 and is considered the gynecological equivalent of transurethral resection syndrome.^1^ OHIA occurs by absorption of the fluid distension medium used during operative hysteroscopy in an exposed vascular bed. A commonly used distension medium in hysteroscopy is 1.5% glycine solution due to its favorable optical and conductive properties. However, 1.5% glycine is hypo-osmolar (200 milliosmole [mOsm)] per kilogram [kg] of water), and absorption of volumes greater than 500 milliliters (mL) have been associated with hyponatremia and cerebral edema.^2^

## CASE REPORT

A 47-year-old female presented by ambulance from an outpatient ambulatory surgical center to the ED secondary to hypoxia, coughing up pink, frothy sputum and with mental status changes. The patient was noted to have a medical history of recurrent uterine fibroids despite two prior hysteroscopic myomectomies over the previous year. She underwent a hysteroscopic myomectomy in an outpatient ambulatory surgical setting, with 1.5% glycine used as distension medium. Intraoperative monitoring of inflow volume of glycine distension medium and collected fluid showed an initial calculated fluid deficit of 600 mL. Repeat measurement 15 minutes later showed the calculated fluid deficit was 2700 mL, at which time the procedure was stopped due to concern for rapid intravascular absorption and the patient was taken to the recovery area. The entire procedure was reported to have lasted less than 30 minutes.

After being brought to the recovery area the patient was given two milligrams (mg) morphine intravenously. She became more and more dyspneic over the next several minutes and began coughing up pink, frothy sputum. She was not complaining of nausea, vomiting or headache. Lung auscultation showed decreased breath sounds in all fields, most prominently at the bases. She was noted to be hypoxic with an oxygen saturation of 82% and was placed on 15 liters per minute (L/min) oxygen by nonrebreather with improvement in oxygen saturation to 98%. She was also treated with two puffs of an albuterol inhaler, intravenous (IV) furosemide 40 milligrams (mg), hydrocortisone 50 mg, and 600 mL of 0.9% saline. She was then transported by ambulance to the ED.

Vital signs on arrival showed blood pressure 99/49 millimeters mercury, respiratory rate 17 breaths/min, heart rate 72 beats/min with oxygen saturation 97% on 15 L/min supplemental oxygen by non-rebreather mask. Attempt at weaning oxygen to 12 L/min was accompanied by oxygen desaturation. Auscultation of the chest on arrival was notable for decreased breath sounds in the lower lung fields bilaterally. The patient was noted to be somewhat somnolent and confused but was easily roused and oriented to person, place and time, with a Glasgow Coma Scale (GCS) score of 12. She had received an additional 100 mL of 0.9% saline during transport for a total of 700 mL prior to arrival. Workup in the ED was significant for serum sodium level of 125 micromoles (mmol)/L (135–145 mmol/L).

**Image 1:**
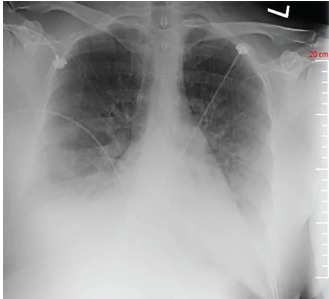
Anterior-posterior chest radiograph demonstrating findings consistent with fluid overload.

Other mild electrolyte abnormalities included serum chloride level of 96 mmol/L (98–110 mmol/L), serum bicarbonate level of 20 (22–32 mmol/L) and serum calcium level of 8.2 mmol/L (8.4–10.4 mmol/L). Chest radiograph showed pulmonary edema, vascular congestion, and bilateral small pleural effusions (Image 1). Computed tomography (CT) of the head was consistent with mild cerebral edema (Image 2). The patient received another 150 mL of 0.9% saline in the ED. Repeat electrolyte measurement two hours after arrival showed serum sodium of 130 mmol/L at which point the IV fluids were stopped. Her mental status had significantly improved to a GCS score of 15, and she was able to maintain an oxygen saturation of 99% on 5 L/min supplemental oxygen by nasal cannula.

She was admitted to the surgical intensive care unit where she was monitored overnight and had electrolyte checks every four hours. A repeat chest radiograph the next morning showed complete resolution of the pulmonary edema and she was saturating 100% on room air. Her serum sodium continued to trend upward and was noted to be 141 mmol/L in the afternoon of the day after arrival. She was discharged from the hospital on postoperative day one.

**CPC-EM Capsule**

What do we already know about this clinical entity?

This is an iatrogenic condition where large-volume, rapid intravascular absorption of operative distension fluid causes acute hyponatremia and fluid overload.

What makes this presentation of disease reportable?

This case highlights a severe presentation of an iatrogenic disease state not commonly treated by emergency physicians (EP).

What is the major learning point?

As ambulatory surgical centers grow in number, EPs will more often care for patients presenting with critical illness related to perioperative complications.

How might this improve emergency medicine practice?

EPs should have a high suspicion for acute hyponatremia with volume overload in patients presenting with altered mental status and respiratory distress after hysteroscopy.

## DISCUSSION

During operative hysteroscopy the use of a fluid medium is used to distend the uterine tissue to allow for optimal visualization. Resectoscopes used in these procedures were initially developed using monopolar current, which necessitated the use of non-electrolyte containing solutions such as 1.5% glycine, 5% dextrose with water, 3% sorbitol, 5% mannitol and 32% Dextran 70 solution. With the development of resectoscopes using bipolar current the use of isotonic electrolyte solutions such as Ringer’s lactate or 0.9% saline may be used as distension media.^3^ Systemic absorption of some of the distension fluid medium is expected, with the average amount of fluid absorbed during operative hysteroscopy cases being approximately 400–600 mL.^4^ Intravascular absorption of fluid is driven by (1) increasing the hydrostatic pressure gradient between the distension fluid and the vasculature, and (2) increasing the surface area of vascular beds exposed to the distension fluid. Increased operative time increases the risk of absorbing larger amounts of fluid. Among hysteroscopic procedures, myomectomies and resections of uterine septa are at higher risk for increased fluid absorption.^5^

Some of the commonly used distension media with monopolar resectoscopy, such as 1.5% glycine solution or 3% sorbitol solution, are hypotonic in addition to being electrolyte free. Intravascular absorption of large amounts of these solutions leads to hypervolemia with dilutional hyponatremia. Using 0.9% saline as distension medium during hysteroscopy with a bipolar resectoscope is not associated with hyponatremia, although there have been reported cases of fluid overload and pulmonary edema.^6^,^7^ There is no exact amount of fluid at which point patients will develop symptoms. Professional society recommendations state that surgeries should be halted once the calculated fluid deficit shows absorption of 1000 mL of hypotonic distension medium in healthy patients or 750 mL in patients with cardiac disease or renal insufficiency. One small study found that nine out of ten patients who experienced intravascular absorption of 1000 mL of 1.5% glycine solution had findings of cerebral edema on head CT. This was accompanied by a 10 mmol/L or more drop in serum sodium levels.^2^,^3^

OHIA syndrome can present with myriad signs and symptoms. Manifestations may include nausea, vomiting, headache, weakness, pulmonary edema, acute respiratory distress syndrome, laryngeal edema, cerebral edema, hyponatremia, hypocalcemia, diffuse intravascular coagulation and rhabdomyolysis.^1^,^2^,^4^,^8^,^9^ This condition is not uncommon and is usually transient and mild in severity; however, it can be life-threatening.^10^ Additionally, premenopausal women who develop postoperative hyponatremic encephalopathy were found in one study to be 25 times more likely to die or develop permanent neurologic sequelae when compared to men and postmenopausal women with hyponatremic encephalopathy. This is attributed to differences in sex hormone influence on sodium pump function in the central nervous system.^11^

Treatment will vary based on symptoms and severity. If an electrolyte-containing isotonic solution such as 0.9% saline was used then the patient will most likely present with symptoms consistent with volume overload, and treatment will center on optimization of respiratory status with supplemental oxygen, non-invasive positive pressure ventilation or intubation as needed and correction of hypervolemia with loop diuretics. If the distension medium was an electrolyte-free hypotonic solution, as was reported in this case, then patients will likely present with electrolyte disturbances in addition to the hypervolemia. As hyponatremia is the most common electrolyte abnormality seen, clinicians should maintain a high degree of suspicion if a patient presents after operative hysteroscopy with neurologic symptoms. Symptomatic and/or severe acute hyponatremia (serum sodium <120 mmol/L) may be treated with 3% hypertonic saline as a 100 mL bolus infused over 10 minutes. This may be repeated up to three times as needed to increase the serum sodium by 4–6 mmol/L to prevent herniation. Patients with less severe presentations may be treated with a slow infusion of 3% hypertonic saline (05.–2 mL/kg/hour).^12^ Alternatively, these patients may be treated with 0.9% saline and loop diuretics. Consultation with a nephrologist or a critical care specialist is recommended. Monitoring of electrolytes every two to four hours is also advised.

Ambulatory surgical centers performed an increasing percentage of all outpatient surgeries in the United States between 2001–2010.^13^ The case presented here highlights some of the challenges with diagnosing and managing these uncommon but severe postoperative syndromes. When symptoms began to manifest in the postoperative period the patient was given supplemental oxygen as well as a dose of furosemide. However, as she was in an ambulatory surgical center, access to diagnostic and therapeutic capabilities was limited. She was started on an infusion of 0.9% saline in a timely manner in the postoperative period. However, a serum sodium level was not measured until she arrived at the ED at which point she had already received 700 mL of 0.9% saline.

This delay in measuring serum sodium does not appear to have adversely influenced the outcome for this patient, but it is unclear whether she would have met criteria for the initiation of hypertonic saline infusion that are outlined in the treatment guidelines.^3^,^12^ Hypertonic saline may have also been helpful in this patient as her severe pulmonary edema could have worsened with higher volumes of 0.9% saline compared to lower volumes of 3% saline that could have been used to correct her hyponatremia if the circumstances were more ideal. Regardless, with limited data the patient was given therapeutic interventions prior to arrival at the ED and she recovered well.

## CONCLUSION

This report highlights a not uncommon entity with an uncommon severity that is familiar to gynecologists and anesthesiologists but is not frequently encountered by emergency providers. Emergency providers should recognize the need to rapidly correct underlying hyponatremia as this can have devastating consequences for patients, in particular premenopausal women.

## Figures and Tables

**Figure 2:**
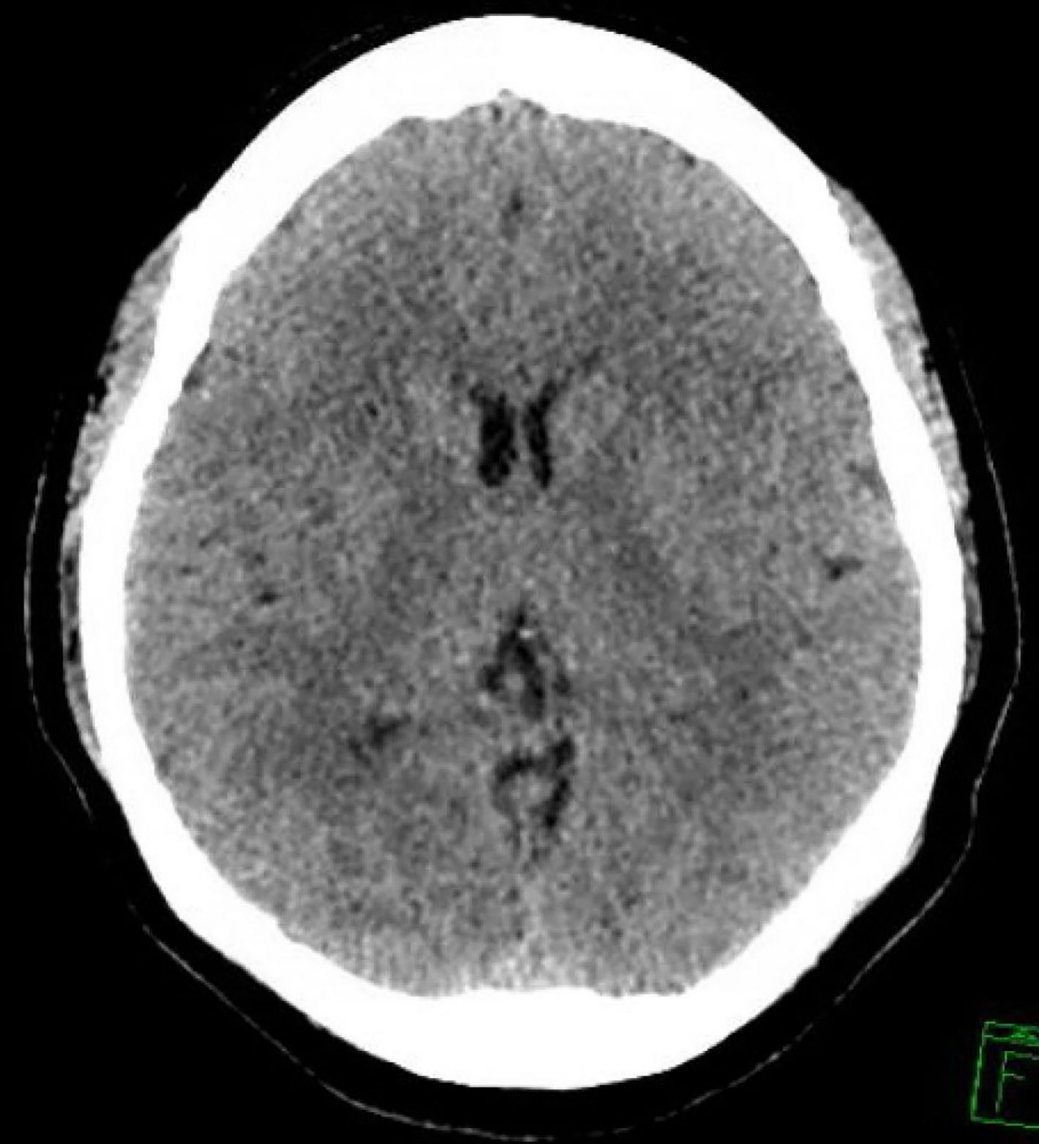
Computed tomography axial image demonstrating mild cerebral edema.
